# An Efficient *Agrobacterium*-Mediated Transformation Method for Hybrid Poplar 84K (*Populus alba* × *P. glandulosa*) Using Calli as Explants

**DOI:** 10.3390/ijms23042216

**Published:** 2022-02-17

**Authors:** Shuang-Shuang Wen, Xiao-Lan Ge, Rui Wang, Hai-Feng Yang, Yu-E. Bai, Ying-Hua Guo, Jin Zhang, Meng-Zhu Lu, Shu-Tang Zhao, Liu-Qiang Wang

**Affiliations:** 1 State Key Laboratory of Tree Genetics and Breeding, Key Laboratory of Tree Breeding and Cultivation of the State Forestry Administration, Research Institute of Forestry, Chinese Academy of Forestry, Beijing 100091, China; 15501131025@163.com (S.-S.W.); gedalan@126.com (X.-L.G.); sorry741786616@163.com (R.W.); sakulaguo@126.com (Y.-H.G.); lumz@caf.ac.cn (M.-Z.L.); shutang@caf.ac.cn (S.-T.Z.); 2 College of Forestry, Inner Mongolia Agricultural University, Hohhot 010019, China; haifeng@imau.edu.cn (H.-F.Y.); baiyue@imau.edu.cn (Y.-E.B.); 3 State Key Laboratory of Subtropical Silviculture, College of Forestry and Biotechnology, Zhejiang A&F University, Hangzhou 311300, China; zhang007jin@163.com; 4 Co-Innovation Center for Sustainable Forestry in Southern China, Nanjing Forestry University, Nanjing 210037, China

**Keywords:** *Agrobacterium*, hybrid poplar, callus, plant transformation, regeneration

## Abstract

A highly efficient *Agrobacterium*-mediated transformation method is needed for the molecular study of model tree species such as hybrid poplar 84K (*Populus alba* × *P. glandulosa* cv. ‘84K’). In this study, we report a callus-based transformation method that exhibits high efficiency and reproducibility. The optimized callus induction medium (CIM1) induced the development of calli from leaves with high efficiency, and multiple shoots were induced from calli growing on the optimized shoot induction medium (SIM1). Factors affecting the transformation frequency of calli were optimized as follows: *Agrobacterium* concentration sets at an OD_600_ of 0.6, *Agrobacterium* infective suspension with an acetosyringone (AS) concentration of 100 µM, infection time of 15 min, cocultivation duration of 2 days and precultivation duration of 6 days. Using this method, transgenic plants are obtained within approximately 2 months with a transformation frequency greater than 50%. Polymerase chain reaction (PCR), reverse transcription-PCR (RT-PCR) and β-galactosidase (GUS) histochemical staining analyses confirmed the successful generation of stable transformants. Additionally, the calli from leaves were subcultured and used to obtain new explants; the high transformation efficiency was still maintained in subcultured calli after 6 cycles. This method provides a reference for developing effective transformation protocols for other poplar species.

## 1. Introduction

Forests cover 30% of the Earth’s surface and provide natural and societal services, such as climate regulation, carbon storage and timber supply [1]. Forest trees have many specific characteristics that distinguish them from herbaceous plants, such as secondary growth, perennial growth and seasonal adaptation [2]. However, the underlying mechanisms of these characteristics are poorly studied. Traditional genetic methods, including crossing and mutagenesis, to disseminate these biological processes are difficult because of the long lifespan of tree species [3]. Therefore, forward genetics is required to study tree biology using model tree species such as *Populus* [4]. Poplars are deciduous trees of the Salicaceae family. More than 52 species are distributed in temperate regions of the Northern Hemisphere [5]. Poplars can attain reproductive maturity within 6 years and provide large-scale sustainable plantations with vast opportunities for molecular selection and breeding [6]. A small genome size, rapid juvenile growth, the availability of extensive genomic sequences, genetic maps and, above all, the feasibility of genetic transformation have allowed poplar to become a model tree species for molecular studies [7].

Plant genetic transformation is useful not only for elucidating gene function but also for breeding plant varieties with targeted traits, particularly traits designed to improve trees with a long generation time, which limits the application of traditional breeding techniques used to establish genetic improvements in plants [2]. *Agrobacterium tumefaciens*-mediated transformation is an effective and useful method to introduce foreign DNA into crops for gene function and breeding research [8,9,10,11]. Several reports have documented the improvement of *Agrobacterium*-mediated transformation of poplars [9,11,12,13,14] using different types of explants, such as leaves, petioles, hypocotyls, stem internodes, stem segments, roots, cell suspension cultures and sprouts [7,12,15,16]. However, these methods suffer from relatively low transformation efficiencies (10–30%) or require a long period of time (2–6 months) [8,9,11,17,18,19]. The recent application of the CRISPR/Cas9 (clustered regularly interspaced short palindromic repeats/CRISPR-associated protein 9)-based genome editing method in trees requires an even higher efficiency of transformation to obtain mutants of both alleles for a particular gene, since heterozygotes cannot be used to quickly create homozygous mutants by selfing due to the long juvenile period of trees [20]. This problem is even more pronounced if several genes in a family are to be knocked out to avoid the gene redundancy that is common in trees [21]. Therefore, robust protocols must be developed to increase the genetic transformation of poplar as a model tree for studies on the molecular regulation of biological processes unique to trees.

A stable and high-efficiency system for transforming calli mediated by *Agrobacterium* has been developed in some plant species, such as rice, soybean, cotton, barley and coffee [22,23,24,25]. For instance, the *Agrobacterium*-mediated transformation of calli is universal for stable integration in rice [26,27,28,29,30]. Compared with other transformation methods, it produces low copy number integrations that exhibit stable expression [31]. Therefore, this method could be used in poplar transformation to achieve high efficiency. Protoplast and callus transformation has been reported to introduce DNA into poplar cells via polyethylene glycol (PEG) or electroporation, but the operation of this method is laborious and can lead to abnormal expression [32]. Methods for *Agrobacterium*-mediated transformation of 84K poplar include direct differentiation of adventitious buds or induction of adventitious buds from callus, but the transformation efficiency and the generation time of the two methods are still unsatisfactory [33]. Different parameters should be optimized for *Agrobacterium*-mediated transformation. Factors such as the explant type, plant growth regulator (PGR) concentration, medium composition, optical density of bacterial cells, antibiotic concentration and infection duration play an important role in the efficiency of transformation [34]. In this study, we developed a simple, rapid and high-throughput *Agrobacterium*-mediated transformation system for hybrid poplar 84K (*Populus alba* × *P. glandulosa* cv. ‘84K’) using callus as the explant. Efficient callus induction, shoot induction and *Agrobacterium*-mediated transformation were carefully optimized. The efficiency of genetic transformation reached more than 50%, and transgenic plantlets were produced within 2 months. This simple, rapid and efficient transformation method significantly promotes the manipulation of genes in 84K poplar.

## 2. Results

### 2.1. Establishment of the Rapid and Efficient Callus Induction and Shoot Induction Protocol

**Figure 1:**
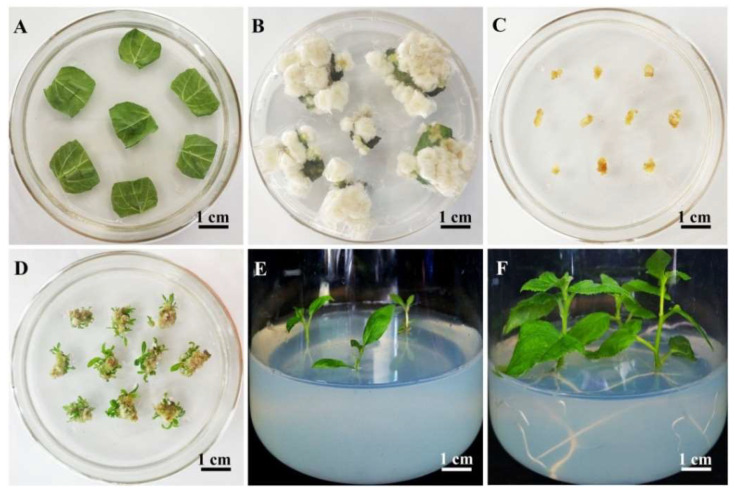
Plant regeneration from 84K poplar calli. (**A**) Leaves of three-week-old plants used for inducing callus formation. (**B**) Calli induced from leaves after 6 weeks on callus induction medium 1 (CIM1). (**C**) Induced calli for shoot induction on shoot induction medium 1 (SIM1). (**D**) Shoots formed from calli after 5 weeks of growth on SIM1. (**E**) Shoots rooting on RM. (**F**) The regenerated plants obtained after 2 weeks of growth on rooting medium (RM).

To develop a highly efficient protocol for *Agrobacterium*-mediated transformation of 84K poplar, four groups of Murashige & Skoog Basal Medium w/Vitamins (MS) (PhytoTech, Lenexa, KS, USA), Lloyd & McCown Woody Plant Basal Medium w/Vitamins (WPM) (PhytoTech, Lenexa, KS, USA), PGR combinations, including 6-furfurylaminopurine (kinetin) (Sigma, St. Louis, MO, USA), 6-benzylaminopurine (6-BA) (Sigma, St. Louis, MO, USA), naphthylacetic acid (NAA) (Sigma, St. Louis, MO, USA) and 2,4-dichlorophenoxyacetic acid (2,4-D) (Sigma, St. Louis, MO, USA) and gelling agents (agar or phytagel) were tested for their effects on callus induction medium (CIM1–4, Table 1). The highest efficiency was observed in CIM1 medium containing WPM (Table 1). However, no significant difference was observed between CIM1 and CIM2, indicating that the use of WPM and MS as the basal medium induced the formation of calli from leaves with similar efficiency (Figure S1A,B). The two types of PGRs induced calli from incised leaves, while a higher induction frequency was obtained in the PGR combination of kinetin and 2,4-D (Figure S1C,D). The CIM1 medium containing WPM, 0.1 mg L^−1^ kinetin, 1.0 mg L^−1^ 2,4-D and phytagel resulted in a higher rate of callus formation, with approximately 97.78% callus formation on incised leaf explants within 2 weeks (Table 1). After 4–6 weeks, these calli were used for shoot induction or genetic transformation (Figure 1B).

The effects of different types of basal medium and gelling agents, with 0.5 mg L^−1^ 6-BA and 0.05 mg L^−1^ NAA, on shoot induction from calli were then evaluated on shoot induction medium (SIM1–3, Table 2). SIM1 medium supplemented with phytagel resulted in a significantly higher frequency (~98.89%) of shoot induction than that supplemented with agar (Table 2; Figure S2), suggesting that phytagel is crucial for the high efficiency of shoot induction.

The actively growing calli (yellowish white and approximately 3–5 mm in diameter) from CIM1 were subcultured on SIM1 (Figure 1C), shoots formed from the callus after approximately 3 weeks; the shoots were used for rooting after 5–6 weeks (Figure 1D). One shoot from each callus was cut and then transferred to rooting medium (RM); the roots appeared within 2 weeks. These shoots, which were approximately 1–2 cm in length, were cut and recultured on RM for 2 weeks; the rooting rate was 100% (Figure 1E,F).

### 2.2. Determination of the Optimal Hygromycin B Concentration for Transformant Selection

**Figure 2:**
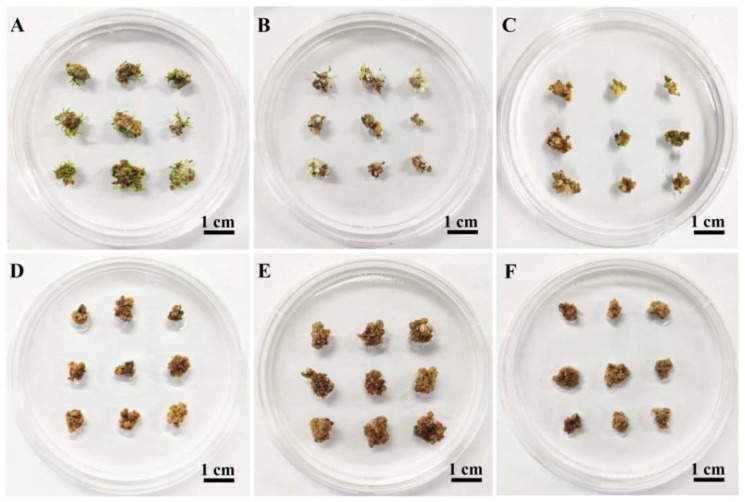
Effect of hygromycin B on shoot induction from 84K calli. (**A**–**F**) The calli were placed on SIM1 supplemented with 200 mg L^−1^ timentin and 0 (**A**), 0.5 (**B**), 1.0 (**C**), 1.5 (**D**), 2.0 (**E**) or 2.5 (**F**) mg L^−1^ hygromycin B. After 5 weeks, the induction of shoots was observed. Three replicates were performed; each replicate contained 30 explants.

Calli were inoculated onto SIM1 supplemented with different concentrations of hygromycin B and 200 mg L^−1^ timentin. Shoot induction was not significantly inhibited by timentin (Figure 2A) but was sensitive to hygromycin B (Figure 2B–F). Compared with SIM1 containing 0 mg L^−1^ hygromycin B, SIM1 supplemented with 0.5 or 1.0 mg L^−1^ hygromycin B resulted in a fewer shoots (Figure 2B,C); shoot induction was completely inhibited by 1.5 to 2.5 mg L^−1^ hygromycin B (Figure 2D–F). Therefore, 1.5 mg L^−1^ hygromycin B was used to select transgenic shoots.

### 2.3. Optimization of the Transformation Procedure

**Figure 3:**
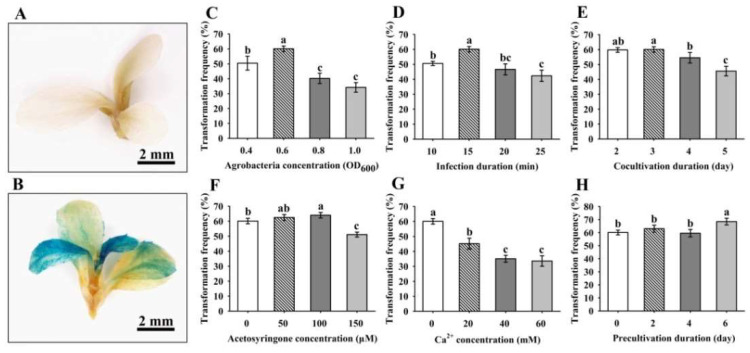
Factors that affect the transformation frequency evaluated from shoots with positive GUS staining. (**A**,**B**) GUS staining in non-transgenic plants (**A**) and transgenic plants (**B**). (**C**–**H**) The *Agrobacterium* concentration, infection duration, cocultivation duration, acetosyringone concentration, Ca^2+^ concentration and precultivation duration were analyzed. Three replicates were performed, and each replicate contained 100 callus explants. The results are presented as the means and standard errors from three independent experiments. Within each variable, values with different letters indicate statistically significant differences at the *p* < 0.05 level.

Based on the results described above, the optimal SIM1 was adopted to test *Agrobacterium*-mediated genetic transformation of 84K. Several factors affecting transformation frequency, including the *Agrobacterium* concentration, infection time, cocultivation duration, acetosyringone (AS) (Sigma, St. Louis, MO, USA) and Ca^2+^ concentrations and preculture duration, were optimized. β-galactosidase (GUS) staining revealed *GUS* expression in hygromycin-resistant shoots but not in non-transgenic shoots (Figure 3A,B). The *Agrobacterium* concentration producing an OD_600_ = 0.6 resulted in the highest efficiency in producing transgenic shoots (Figure 3C). Regarding the infection time, 15 min was the most suitable time for efficient transformation of calli with the *Agrobacterium* culture at a concentration of OD_600_ = 0.6 (Figure 3D). The highest transformation efficiency was obtained after cocultivation for 2 or 3 days; a significant difference was not observed (Figure 3E). The addition of AS to the *Agrobacterium* suspension improved the transformation efficiency, with a high frequency obtained at an AS concentration of 100 µM (Figure 3F). Lower Ca^2+^ concentrations increased, but higher concentrations decreased, the transformation efficiency (Figure 3G). Moreover, 6 days of precultivation on cocultivation medium (CM) before transformation increased the efficiency (Figure 3H).

**Figure 4:**
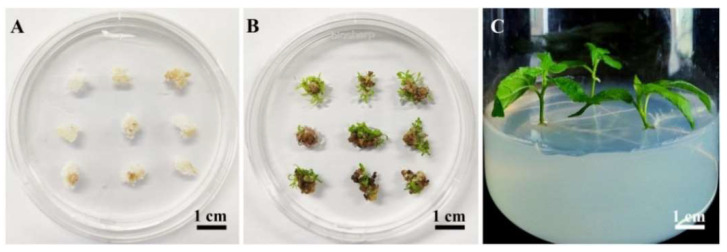
Regeneration of transgenic 84K plants using the optimized *Agrobacterium*-mediated transformation system based on calli. (**A**) Infected calli were cocultivated for 6 days. (**B**) Infected calli were cultivated on shoot induction medium 1 (SIM1) containing timentin and hygromycin B for 5 weeks. (**C**) Putative transgenic shoots were transferred and cultured on rooting medium (RM) supplemented with timentin and hygromycin B for 2 weeks.

Based on the optimized transformation system for 84K poplar, 100 actively growing calli from each of the three biological replicates were inoculated with *Agrobacterium* cells carrying the 35S::GUS binary vector (Figure 4A). Within 5 weeks, 72, 70 and 65 independent hygromycin-resistant shoots were obtained on SIM1 containing hygromycin B (Figure 4B). Later, these resistant shoots with lengths of 1–2 cm were cut from calli and cultured on RM with hygromycin B for 2 weeks, and 62, 59 and 56 shoots rooted, resulting in healthy seedlings (Figure 4C). Nevertheless, the remaining shoots did not root and grew slowly or died with chlorotic symptoms, suggesting that they might be chimeras.

**Figure 5:**
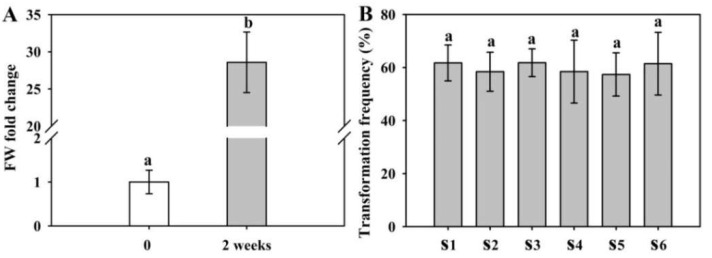
The growth biomass of callus and callus subculture generation affects the transformation frequency of shoots. (**A**) Quantification of the biomass of growing calli. The calli were subcultured on CIM1 for 2 weeks, and the fresh weight (FW) was measured. (**B**) Effect of the callus subculture generation on the transformation efficiency of GUS-positive hygromycin-resistant shoots. S1–S6, Number of subcultured calli. Three replicates were performed; each replicate contained 30 callus explants. The results are presented as the means and standard errors. Within each variable, values with different letters indicate statistically significant differences at the *p* < 0.05 level.

We further investigated whether we could obtain more calli as explants by subculture and use them for transformation. The biomass of calli was increased by approximately 28 times in fresh weight after 2 weeks in one subculture (Figure 5A). In addition, the obtained calli were subcultured at least 6 times; all of them maintained a high transformation efficiency of more than 50% (Figure 5B).

### 2.4. Confirmation of the Transgenic Plants

**Figure 6:**
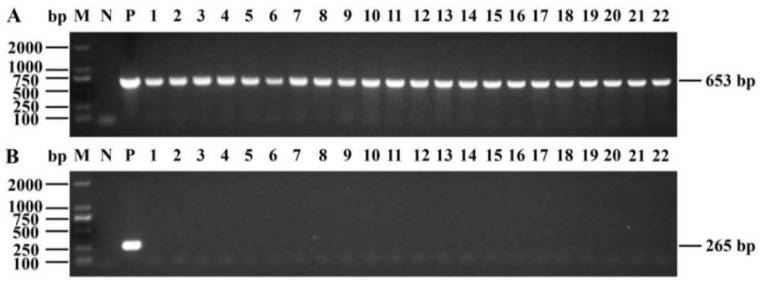
Molecular analyses of the transgenic plants expressing the *GUS* gene. (**A**,**B**) PCR amplification of the *GUS* (653 bp) and *aadA* (265 bp) genes in transgenic lines (22 lines are shown) using genomic DNA as templates. M, 2000 bp DNA marker; N, non-transgenic 84K plants used as a negative control; P, 35S::GUS binary vector used as a positive control; 1–22, the independent transgenic lines displayed bands for *GUS* but not for the *aadA* gene.

**Figure 7:**
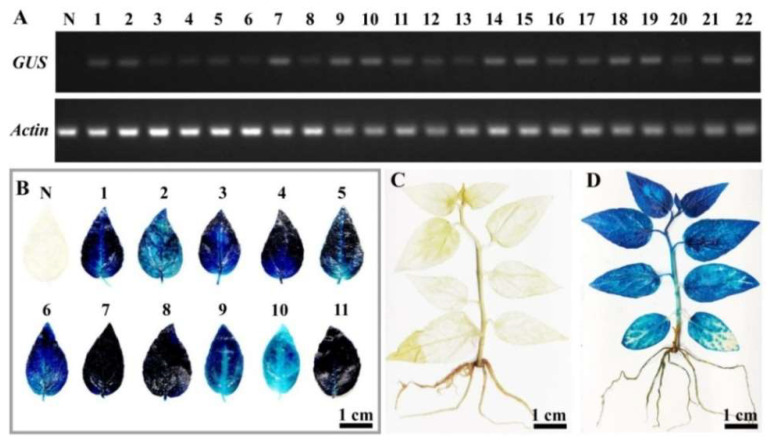
*GUS* expression in transgenic plants. (**A**) RT-PCR analysis of *GUS* transcript levels in non-transgenic plants (N) and transgenic lines (1–22), as shown in Figure 6. *Actin* expression was used as an internal control. (**B**) GUS assay in leaves of non-transgenic (N) and transgenic lines (11 lines are shown). (**C**,**D**) Histochemical staining for GUS activity in regenerated non-transgenic (**C**) and transgenic (**D**) plants. GUS staining was observed in 3-week-old transgenic plants but not in the non-transgenic plants.

To further confirm the insertion of the *GUS* gene into the 84K genome, all putative transgenic lines were examined using PCR analysis of genomic DNA with *GUS*-specific primers. A 653 bp band was detected in all transgenic lines and not in non-transgenic 84K plants (Figure 6A), while the 265 bp band of the *aadA* gene in the pCAMBIA1301 binary vector was not detected in these transformants (Figure 6B), excluding the possibility of *Agrobacterium* cell contamination. RT-PCR analysis further revealed *GUS* gene expression in transgenic seedlings at the transcriptional level (Figure 7A). Furthermore, GUS staining was detected in the regenerating transgenic plants at the protein expression level, whereas no GUS staining was observed in non-transgenic 84K plants (Figure 7B–D). Taken together, these results indicated that *GUS*, a foreign gene, was successfully integrated into the genome of 84K plants and normally expressed in all transgenic poplars tested.

## 3. Discussion

Transgenic technology not only facilitates research on plant molecular biology but also provides a powerful tool for molecular breeding [35,36]. As a model tree species in *Populus*, poplar 84K has been widely used in molecular biology and as an important clone in forestry production [37]. However, low and unstable transformation efficiency prevents its full utilization in both molecular research and breeding. In this study, we established a simple and reliable *Agrobacterium*-mediated transformation system for 84K poplar using calli. The overall short duration of the transformation and plant regeneration process and the high transformation efficiency might facilitate gene manipulation, such as multiple gene editing, in this hybrid poplar.

The type and concentration of PGRs, the sources of carbohydrates and gelling agents are the main factors that affect the results of organogenesis [38]. Plants regenerate their tissues, specific organs, or even entire individuals from explants or some cells, which is achieved quickly by hormonal induction [39]. Organogenesis requires different PGRs for shoot and root induction [38]. Various combinations of auxin and cytokinin have been examined to generate calli and to induce the regeneration of roots and shoots in various species [40]. In rice, calli derived from mature seeds and immature embryos are widely used in *Agrobacterium*-mediated genetic transformation with high transformation frequency [41]. However, few reports have described the genetic transformation of woody plants using calli. Callus induction and shoot induction, which are essential prerequisites for *Agrobacterium*-mediated transformation, were carefully optimized in this study to develop a callus-based transformation method for 84K poplar. Different basal media, PGRs, and gelling agents and their interactions are the key factors to consider for this purpose. Basal media contains different macro- and micronutrients, vitamins, and amino acids that potentially contribute to organogenesis in plants, such as WPM and MS media [42]. We found that both basal WPM and MS media supplemented with the same PGRs induced callus formation from leaves and regenerate shoots, but the frequency of callus induction and shoot induction in culture medium containing WPM was higher than that in MS (Table 1 and Table 2). Compared with MS medium, WPM contains less nitrogen and a lower salt content, which may cause differences in the frequency of callus and shoot formation, as proposed in previous studies [43,44,45]. Phytagel addition induced the formation of a large number of healthy calli (Table 1) with a higher shoot induction frequency than agar (Table 2), making it suitable for the establishment of a high-throughput transformation system. Gelling agents (phytagel or agar) affect the chemical and physical characteristics of the culture medium, such as the diffusion rate of nutrients, elemental and organic impurities and gel strength, which may influence the effect on callus induction and shoot induction [46,47].

*Agrobacterium* is a powerful tool for plant research through the overexpression and/or downregulation of specific genes. However, *Agrobacterium*-mediated transformation is also a complex procedure in which many factors potentially affect its efficiency. Factors such as the optical density of *Agrobacterium*, antibiotic-mediated *Agrobacterium* death, concentration of AS, inoculation duration and cocultivation duration affect the efficiency of *Agrobacterium*-mediated transformation [38,48]. The duration of infection and coculture affect the transformation efficiency by altering the interaction between *Agrobacterium* and plant cells [17,49]. In some *Populus* species, the optimal optical density of the *Agrobacterium* concentration ranges from an OD_600_ of 0.3 to 1.0 [7,11,15,17]. In this study, the highest frequency was achieved when the explants were infected with *Agrobacterium* at an OD_600_ = 0.6 for 15 min and cocultured for 2–3 days, while higher concentrations led to a lower transformation frequency (Figure 3C–E). The transcript levels of the virulence (*vir*) genes affect the transformation efficiency of *Agrobacterium* strains by enhancing *Agrobacterium* infection of wound segments [48]. AS induces *vir* gene expression and promotes *Agrobacterium* to transfer T-DNA into the plant genome [16]; some studies have shown that the transformation efficiency increases in the presence of AS in cocultivation medium [7,9,16,17]. In the current experiment, the addition of 100 µM AS to the *Agrobacterium* suspension, cocultivation and precultivation improved the transformation frequency of 84K (Figure 3F). Ca^2+^ increases the *Agrobacterium*-mediated transformation efficiency in rice [50]. However, at concentrations of 20–60 mM, Ca^2+^ significantly decreased the *Agrobacterium*-mediated transformation of calli, indicating that a higher concentration of Ca^2+^ was not suitable for callus transformation (Figure 3G). Precultivation promotes the proliferation of the callus to provide numerous cells as potential targets for transformation [51]; 6 days of precultivation resulted in the highest transformation efficiency for calli (Figure 3H). A threshold exists for the applied parameters in *Agrobacterium*-mediated gene transformation. Levels and concentrations greater than the optimum value will exert an inhibitory effect. For example, a higher *Agrobacterium* concentration, longer infection duration, coculture duration, and higher AS and Ca^2+^ concentrations significantly reduced the regeneration efficiency. The efficiency of *Agrobacterium*-mediated transformation depends on the interactive effects of several pivotal parameters. Based on the optimized transformation system for 84K poplar, a greater than 50% transformation frequency was achieved for 84K poplar using this callus-based transformation method.

Although the leaf disc transformation-regeneration method has been widely used for poplar transformation [2,8,15,52], it suffers from several limitations. For instance, a large number of leaves are required as explants for genetic transformation, increasing the difficulty of performing large-scale experiments [53]. According to published studies, different genotypes exhibit variable genetic transformation frequencies and within different durations as follows: 20% for *P*. *nigra var*. *italica* in 3 months [18], 16% for *P*. *tremuloides* in 3–4 months [19], 30% for *P*. *davidiana* Dode × *P. bollena* Lauche in 2–3 months [8] and 26.7% for *P*. *trichocarpa* (genotype Nisqually-1) in 2 months [17]. Nevertheless, the general low efficiency and reproducibility of these genotypes might lead to difficulty in multiple gene transformation and editing. In the present study, the method using callus transformation was suitable and useful to transform genes into 84K poplar, with high efficiency (>50%), simplicity (with 2 months), and reproducibility. In addition, the callus can be subcultured to increase the biomass, and a large number of explants can be obtained at any time, suggesting that the method is suitable for routine large-scale transformation using *Agrobacterium*. More importantly, although the callus was subcultured 6 times, it still maintained a stable and high transformation efficiency. Based on these results, this method holds potential for the establishment of a large-scale mutant library in poplar through T-DNA insertions, which is difficult to achieve using the leaf disc transformation-regeneration method.

## 4. Materials and Methods

### 4.1. Plant Materials

Hybrid poplar 84K (*Populus alba* × *P*. *glandulosa* cv. ‘84K’) was used to obtain explants for the transformation study. Stems of 84K were propagated from microcuttings in bottles and cultured on rooting medium (RM) containing half-strength MS (1/2 MS), 30 g L^−1^ sucrose, 5 g L^−1^ agar, 0.05 mg L^−1^ indolebutyric acid (IBA) (Sigma, St. Louis, MO, USA) and 0.02 mg L^−1^ naphthylacetic acid (NAA) in a phytotron (25 ± 1 °C temperature, 60 ± 5 μmol photons m^−2^ s^−1^, 16 h/8 h light/dark photoperiod and 55 ± 5% relative humidity).

### 4.2. Optimization of Callus Induction Medium

The 3rd to 5th fully expanded leaves of 3-week-old 84K plantlets were cut with a scalpel; the incised leaf explants were cultured on four groups of callus induction medium (CIM1-4) containing 20 g L^−1^ sucrose and 0.5 g L^−1^ 4-morpholineethanesulfonic acid (MES) (Sigma, St. Louis, MO, USA) that was supplemented with various combinations of basal medium, including MS, WPM, PGRs, agar and phytagel (Table 1). 6-BA, 2,4-D, kinetin and NAA were used at different concentrations as PGRs in the basal medium. The pH of the medium was adjusted to 5.90 with 0.1 mol L^−1^ NaOH or 0.1 mol L^−1^ HCl and then autoclaved at a pressure of 1.1 kg cm^−2^ (121 °C) for 20 min. Four groups of medium combinations among basal medium, PGR and gelling agent were tested for their effects on callus induction. After 4 weeks of culture in the dark at 25 °C, the effect of each treatment on callus induction was surveyed. The mean number of calli per medium indicates the number of leaves that regenerated calli, and the induction frequency of calli was calculated as follows: the number of leaf explants regenerating calli/total number of cultured leaf explants. Each treatment contained 30 explants, and three replicates were performed. The callus was subcultured on fresh CIM medium every 2 weeks and used for *Agrobacterium*-mediated transformation.

### 4.3. Optimization of Shoot Induction Medium

Three groups of shoot induction media (SIM1-3) containing different basal media (WPM or MS), 20 g L^−1^ sucrose, 0.5 g L^−1^ MES, and 5.0 g L^−1^ gelling agents (agar or phytagel) along with 0.05 mg L^−1^ NAA and 0.5 mg L^−1^ 6-BA were used to culture friable and yellow granular calli to evaluate their ability to induce shoots (Table 2). The shoot induction media were adjusted to a pH of 5.90 and then autoclaved at a pressure of 1.1 kg cm^−2^ (121 °C) for 20 min. Thirty individual calli were used for each treatment; the experiments were performed for three replicates. The mean number of shoots per medium indicates the number of calli that regenerated shoots. The number of shoots was calculated, and the induction frequency of shoots was measured (the number of explant regenerating shoots/total number of cultured callus explants).

### 4.4. Optimizing the Hygromycin B Concentration for the Selection of Transformants

For the optimization of the hygromycin B concentration in shoot induction selection, the callus was placed on optimal SIM that contained timentin (200 mg L^−1^) and different concentrations of hygromycin B (0, 0.5, 1.0, 1.5, 2.0 or 2.5 mg L^−1^). After 5 weeks of culture in a growth chamber (25 ± 1 °C, 16/8 h light/dark), shoot induction was investigated. All experiments were performed with three independent replicates, and each replicate contained 30 calli.

### 4.5. Transformation of Callus via Agrobacterium Cells

*Agrobacterium* strain GV3101 cells harboring the binary expression vector pCAMBIA1301 containing the β-glucuronidase (*GUS*) reporter gene driven by the cauliflower mosaic virus (CaMV) 35S promoter were used for the genetic transformation of calli. A single colony of *Agrobacterium* carrying the pCAMBIA1301 binary vector was inoculated into 3 mL of liquid Luria-Bertani medium (LB; 5 g L^−1^ sodium chloride, 5 g L^−1^ yeast extract and 10 g L^−1^ tryptone) containing 20 mg L^−1^ rifampicin, 50 mg L^−1^ gentamicin and 50 mg L^−1^ kanamycin and cultured overnight at 28 °C with agitation at 200rpm. Then, 0.2mL of the bacterial cultures was transferred to 50 mL of fresh liquid LB containing appropriate antibiotics and incubated under the same culture conditions until reaching an OD_600_ of 0.4–1.0. The cells were collected by centrifugation at 3500 rpm for 15 min and suspended in 100 mL of liquid CIM1 containing 100 µM acetosyringone (AS) adjusted to pH 5.60 for transformation.

### 4.6. Evaluation of the Factors Affecting the Transformation Frequency

Rapid-growing and well-separated calli were used for transformation. The following six factors were evaluated for transformation frequency: *Agrobacterium* concentration (OD_600_ = 0.4, 0.6, 0.8 and 1.0), infection duration (10, 15, 20 and 25 min), cocultivation duration (2, 3, 4 and 5 days), AS concentration (0, 50, 100 and 150 µM) and Ca^2+^ concentration (0, 20, 40 and 60 mM) of the *Agrobacterium* suspension with the callus, in addition to the precultivation duration (0, 2, 4 and 6 days) of the cut callus (from a large mass) on CIM. Calli of approximately 1 cm^3^ in size were submerged in the *Agrobacterium* suspension, slightly shaken periodically, and then blotted with sterile filter paper to remove the excess bacterial suspensions. The infected calli were then placed on cocultivation medium (CM) containing WPM, 20 g L^−1^ sucrose, 0.5 g L^−1^ MES and 100 µM AS. After cocultivation in a dark incubator at 25 °C, the explants were collected in sterile bottles, washed with sterile water at least four times, and submerged in 200 mg L^−1^ timentin in water for 30 min to control the bacteria. The explants were then transferred into optimal SIM containing 200 mg L^−1^ timentin and 1.5 mg L^−1^ hygromycin B to induce the growth of hygromycin-resistant shoots. After approximately 5 weeks of culture in a growth chamber at 25 °C with a 16/8 h light/dark cycle, one resistant shoot from each callus with a length of 1–2 cm was cut and rooted on RM supplemented with 1.5 mg L^−1^ hygromycin B. Each treatment contained 100 explants; three replicates were performed for each treatment. All hygromycin-resistant shoots were detected using GUS histochemical staining; the transformation frequency was calculated as follows: the number of explants regenerating GUS-positive shoots/total number of cultured callus explants.

### 4.7. PCR and RT-PCR Analyses

After growth on RM for 3 weeks, the leaves of transgenic and non-transgenic plants were collected and used for PCR analysis. Genomic DNA was extracted from the leaves of transgenic and non-transgenic plants that were grown on RM for 3 weeks. Using these genomic DNAs as templates, the *GUS* gene fragment (653 bp) was amplified using a primer pair (5′-CGATGTCACGCCGTATGT-3′ and 5′-CGTAAGGGTAATGCGAGGT-3′). In addition, the DNA fragment (265 bp) of the kanamycin resistance gene *aadA* in the pCAMBIA1301 binary vector was used to assess the possible *Agrobacterium* contamination of the regenerated plants using a gene-specific primer pair (5′-ACGCAGAAGGCAATGTCAT-3′ and 5′-ACAGCCGCTTAGCCGAAT-3′). *GUS* expression was quantified using reverse transcriptional PCR (RT-PCR) with primers 5′-TCTACTTTACTGGCTTTGGTCG-3′ and 5′-CGTAAGGGTAATGCGAGGTAC-3′ [54]. *PagActin* was selected as a reference gene using a specific primer pair (5′-AAACTGTAATGGTCCTCCCTCCG-3′ and 5′-GCATCATCACAATCACTCTCCGA’) [55].

### 4.8. GUS Staining Assay

One-month-old non-transgenic 84K and transgenic plants grown on 1/2 MS solid medium in the phytotron were used for GUS staining and analysis. GUS histochemical staining was performed using the method described in a previous study [56]. After GUS staining, 70% (*v*/*v*) ethanol was used to remove the chlorophyll. The chlorophyll-free stained plants were visualized under a microscope [56].

### 4.9. Statistical Analysis

SPSS 18.0 (Chicago, IL, USA) was used for analyses of all data. The value *p* < 0.05 was considered statistically significant.

## 5. Conclusions

**Figure 8:**
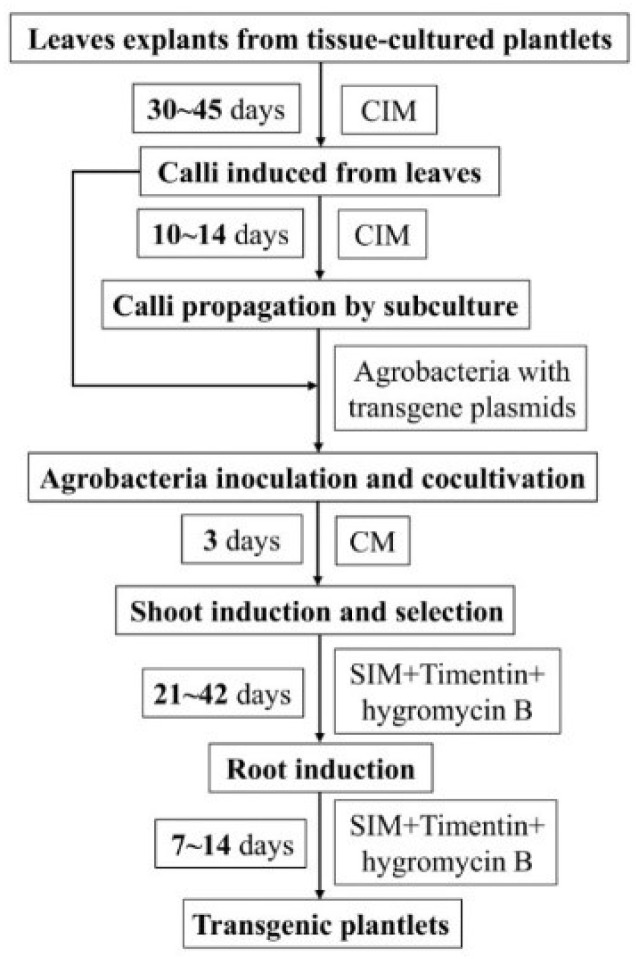
Stepwise protocol for transforming 84K plants using calli as the explant. CIM, callus induction medium; CM, cocultivation medium; SIM, shoot induction medium; RM, rooting medium.

In this study, we established a rapid, convenient and high-throughput *Agrobacterium*-mediated transformation system for 84K poplar using calli as explants (Figure 8). The method holds three major advantages compared to commonly used protocols. First, a large number of transformed shoots are induced from calli; thus, high throughput, simplicity and convenience are achieved. Second, a large number of calli are subcultured as explants, which maintain a stable and high transformation efficiency. Third, the method provides a greater than 50% transformation frequency, and transgenic plants are obtained in large quantities in approximately 2 months. Therefore, this basic tool might promote molecular research and molecular breeding in poplar. Moreover, based on this protocol, we will use the CRISPR/Cas9 system to develop highly efficient target gene mutagenesis in this model tree in the future; future research is needed to explore better gene transformation protocols and to assess the T-DNA integration after gene transformation in poplar.

## Figures and Tables

**Table 1:** Effect of different culture media on callus induction from 84K leaves.

Culture Medium	Basal Medium	2,4-D (mg/L)	Kinetin (mg/L)	NAA (mg/L)	6-BA (mg/L)	Gelling Agent	Number of Leaf Explants	Mean Number of Calli per Medium	Induction Frequency (%)
CIM1	WPM	1.0	0.1	0	0	Phytagel	30	29.33 ± 0.33 a	97.78 ± 1.11 a
CIM2	MS	1.0	0.1	0	0	Phytagel	30	28.33 ± 0.88 a	94.44 ± 2.94 a
CIM3	MS	0.2	0	0.4	0.4	Phytagel	30	24.67 ± 0.88 b	82.22 ± 2.94 b
CIM4	MS	0.2	0	0.4	0.4	Agar	30	22.00 ± 1.00 b	73.33 ± 3.33 b

Different letters indicate significantly different mean values at the 0.05 probability level.

**Table 2:** Effect of different culture media on shoot induction from 84K calli.

Culture Medium	Basal Medium	NAA (mg/L)	6-BA (mg/L)	Gelling Agent	Number of Callus Explants	Mean Number of Shoots per Medium	Regeneration Frequency (%)
SIM1	WPM	0.05	0.5	Phytagel	30	29.67 ± 0.33 a	98.89 ± 1.11 a
SIM2	WPM	0.05	0.5	Agar	30	5.00 ± 0.58 b	16.67 ± 1.92 b
SIM3	MS	0.05	0.5	Agar	30	1.67 ± 0.33 c	5.56 ± 1.11 c

Different letters indicate significantly different mean values at the 0.05 probability level.

## Supplementary Materials

The following supporting information can be downloaded at: https://www.mdpi.com/article/10.3390/ijms23042216/s1.
