# Supplementary material for: An Efficient Agrobacterium-Mediated Transformation Method for Hybrid Poplar 84K (Populus alba × P. glandulosa) Using Calli as Explants
Source: Int J Mol Sci. 2022 Feb 17;23(4):2216. doi: 10.3390/ijms23042216 (PMC8879841; doi:10.3390/ijms23042216)
Supplement: Supplementary file 1 [file ijms-23-02216-s001.zip › ijms-1552478-supplementary.pdf]

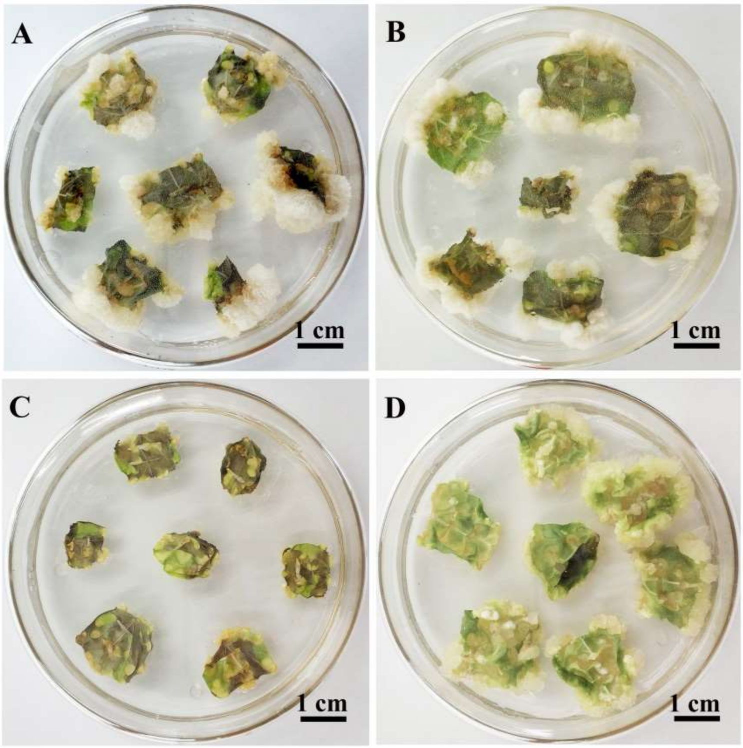

Figure S1. Effects of different culture media on callus induction from 84K leaves. The leaves were placed on CIM1 (**A**), CIM2 (**B**), CIM3 (**C**), and CIM4 (**D**). After 6 weeks, the induction of callus regeneration was observed. Three replicates were performed, and each replicate contained 27 explants.

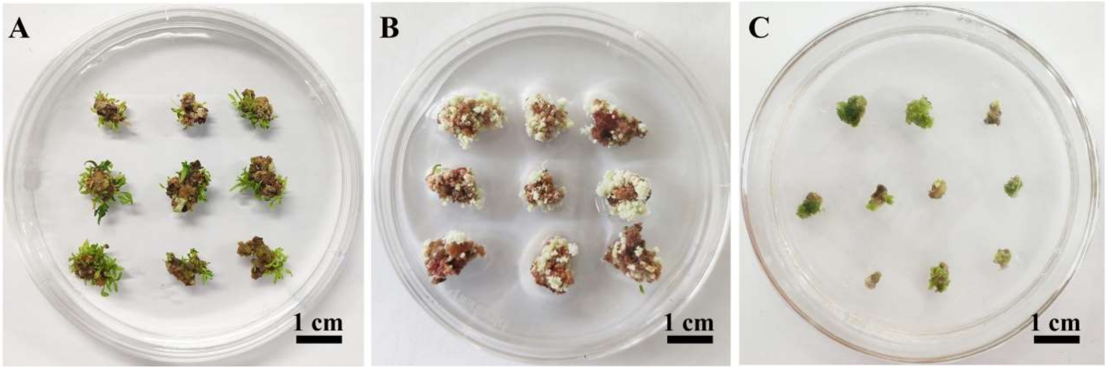

Figure S2. Effects of different culture media on shoot induction from 84K calli. The calli were placed on SIM1 (**A**), SIM2 (**B**), and SIM3 (**C**). After 5 weeks, the induction of shoot growth was observed. Three replicates were performed, and each replicate contained 27 explants.
